# Experimental Demonstration of Surface Plasmon Polaritons Reflection and Transmission Effects

**DOI:** 10.3390/s19214633

**Published:** 2019-10-24

**Authors:** Lei Zheng, Urs Zywietz, Andrey Evlyukhin, Bernhard Roth, Ludger Overmeyer, Carsten Reinhardt

**Affiliations:** 1 Hannover Centre for Optical Technologies, Leibniz Universität Hannover, Nienburger Straße 17, 30167 Hannover, Germany; 2 Cluster of Excellence PhoenixD (Photonics, Optics, and Enigineering-Innovation Across Disciplines), 30167 Hannover, Germany; 3 Laser Zentrum Hannover e.V., Hollerithallee 8, 30419 Hannover, Germany; 4 Institute of Quantum Optics, Leibniz Universität Hannover, Welfengarten 1, 30167 Hannover, Germany; 5 Institute of Transport and Automation Technology, Leibniz Universität Hannover, An der Universität 2, 30823 Garbsen, Germany; 6 Hochschule Bremen, Neustadtswall 30, 28199 Bremen, Germany

**Keywords:** surface plasmon polaritons, surface waves manipulation, microscope projection photolithography, plasmon leakage radiation, plasmonic sensing

## Abstract

Special integrated photonic surface structures composed of a dielectric semicircle ridge and a dielectric block placed on a metal substrate are proposed for the investigation of surface plasmon polariton (SPP) reflection and transmission effects. A fabrication method called microscope projection photolithography was employed for the preparation of the structures. Leakage radiation microscopy was applied for the excitation and observation of surface plasmon polaritons (SPPs). It was observed that SPPs exhibit a remarkable decrease in intensity when impinging onto the rectangular dielectric block. Nevertheless, the transmitted wave out of the dielectric block was always observable. The propagation behavior of both the reflected waves at two boundaries (air/dielectric and dielectric/air) and the transmitted wave inside the dielectric block were demonstrated for different SPP incident conditions. The variation of the angles of reflection and transmission with respect to the incident angle was analytically and experimentally investigated. An agreement between the calculated results and the experimental results was obtained. Our findings might allow for novel applications in sensing and analytics once the structures will be functionalized.

## 1. Introduction

Surface plasmon polaritons (SPPs) are highly confined electromagnetic surface waves that propagate along the interface of a dielectric and a metal [1], with an electric field component parallel to the propagation direction and exponential decay in the direction perpendicular to the interface [2,3]. This type of propagating surface electromagnetic waves can be generated by means of prism coupling configuration [4,5], near-field excitation [6,7], surface grating coupling [8,9,10], surface roughness [1], surface defects [11] or sharp edges [12], among others. The propagation of SPPs along the interface is a complex phenomenon that combines the micro-electric and macro-optical properties. This provides the opportunity of interfacing with high-speed photonic elements and small-sized electric circuits [13,14,15]. Additionally, SPPs possess the unique properties of strong surface field enhancement and large light localization at the nanoscale. These features have fostered the research interest on plasmonics-based applications in fields like information technology [14], imaging and sensing [16,17,18,19], optoelectronics [20,21,22] and nanophotolithography [23,24,25,26,27,28]. Various optical devices, such as light concentrators [29,30,31,32], waveguides [33,34,35,36], lenses [37,38,39], photodetectors [40,41,42,43,44], heterostructures [45,46,47], metamaterials [48,49,50,51,52], reflectors [53,54] and deflectors [55,56], have been proposed and demonstrated to realize various functionalities through the control of SPP propagation at the subwavelength scale [57,58]. Consequently, the manipulation of SPPs plays an essential role in the performance of plasmonic devices. One fundamental research issue concerning the manipulation is the tuning of the propagation behavior of SPPs at the boundary between two media with different dielectric constants [3]. This behavior mainly includes reflection, transmission, scattering and diffraction. To date, a variety of analytical studies on this issue has been extensively carried out [2,3,13,59,60,61,62,63,64]. However, only a few experiments were performed for investigation of the reflection, scattering and refraction of SPPs, which were mainly realized based on structures such as plane mirrors [65], metal wedges [66] and nanoparticle arrays [67]. Further experimental investigations are still desirable for deeper insight into the complicated propagation behaviors of SPPs at different boundaries, as well as the exploration of potential applications such as sensing.

**Figure 1:**
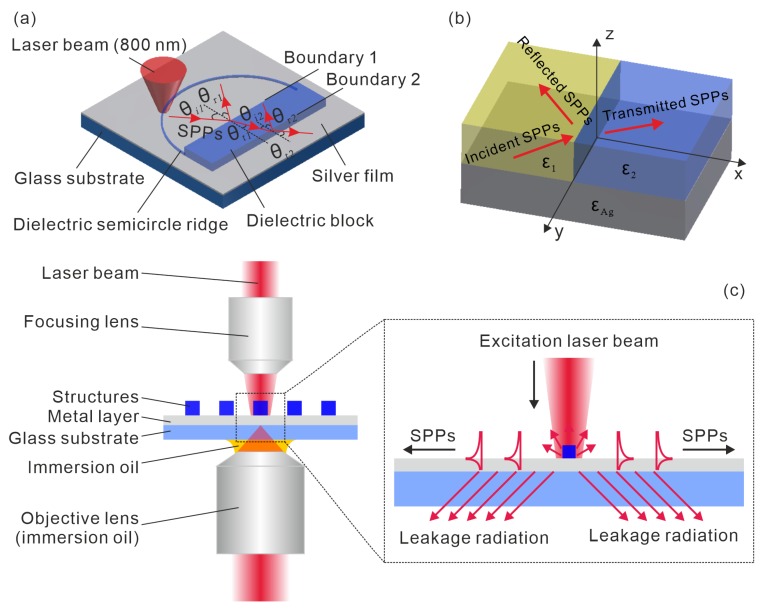
Geometrical illustrations. (**a**) Geometrical representation of the proposed structure. (**b**) Schematic illustration of reflection and transmission of surface plasmon polaritons (SPPs) at the interface of two different homogeneous dielectrics. (**c**) Schematic illustration of generating SPPs by surface defects and visualization principle of propagating SPPs through leakage radiation microscopy (LRM). A detailed description of LRM can be found in ref [69].

In this paper, a novel integrated photonic surface structure (Figure 1a) was created with the purpose of investigating the reflection and transmission effects of propagating SPPs at the interface on two different media. The proposed structure, which was placed on a metal film, is composed of a dielectric semicircle ridge and a dielectric block as illustrated in Figure 1a. The ridge was used as a surface defect to excite SPPs by focusing a laser beam onto it and controlling the incident angle of the SPPs. The dielectric block was used to generate a space with a different dielectric constant from the surrounding air. The commonly used metals for the generation of surface plasmons are silver and gold. In this experiment, silver was used due to its longer SPP propagation length than gold [11]. The reflection and transmission of SPPs take place at the boundary between the air and the dielectric block as illustrated in Figure 1b. Additionally, SPPs can also be scattered into light waves propagating away from the metal surface, which decreases the intensity of the reflected and transmitted SPPs.

As to the preparation of samples, a silver film with the thickness of 60 nm was first spin-coated on a clean glass substrate. For the fabrication of proposed structures, the smoothness of the semicircle ridge and the lateral surface of the dielectric block has to be guaranteed in order to reduce the scattering of SPPs and thus improve the performance of the structure. With this consideration, a technique called microscope projection photolithography (MPP) [68], which is able to implement a fast fabrication process and achieve high structuring quality, was employed for the preparation of the proposed surface structure. Afterwards, leakage radiation microscopy (LRM) [69], which visualizes the propagating SPPs by collecting the waves leaked out of the substrate, was applied to excite and observe SPPs on the fabricated structures. A schematic diagram illustrating the principle of LRM for generating and visualizing SPPs is presented in Figure 1c. A detailed introduction of the system can be found in ref [69]. When the laser beam (the wavelength λ=800 nm) is focused onto the ridge of the proposed structure, SPPs are excited and propagate to both the outer and inner radial directions of the ridge. Along the inner direction, SPPs propagate towards to the dielectric block. The reflection and transmission of SPPs at the boundaries between the air and the dielectric block can be observed using the LRM setup. By moving the laser focus along the semicircle ridge, the incident angle of SPPs striking onto the dielectric block can be varied. The dependence of the angles of reflection and transmission on the incident angle of SPPs is investigated and discussed both analytically and experimentally.

## 2. Theoretical Model

It is known that when a plane wave impinges onto a boundary between two homogeneous media with different dielectric constants, the plane wave can be split into a reflected wave and a transmitted wave, with the reflected wave propagating back to the first medium and the transmitted wave proceeding into the second medium [70]. The propagation directions of the reflected wave and the transmitted wave can be expressed using $\theta_{r}=\theta_{i}$ (the law of reflection) and $k_{t}\sin\theta_{t}=k_{i}\sin\theta_{i}$ (Snell’s law) [70], with $\theta_{i}$ indicating the angle of incidence, $\theta_{r}$ the angle of reflection and $\theta_{t}$ the angle of transmission, $k_{i}$ the wave number of the incident light and $k_{t}$ the wave number of the transmitted light. Both laws are deduced from the boundary conditions, which have to be satisfied for the existence of the reflected and transmitted waves. Therefore, the reflection and transmission of surface plasmon polaritons (SPPs) have to fulfil the boundary conditions at the interface between isotropic media with different dielectric constants.

A geometrical illustration of the reflection and transmission of SPPs at the boundary of two homogeneous media with the dielectric constants $\varepsilon_{1}$ and $\varepsilon_{2}$ is presented in Figure 1b. In this work, the dielectric block is made of ma-N 1405, which is a commercially available photoresist from micro resist technology GmbH and has a dielectric constant $\varepsilon_{2}=2.64$ at the wavelength of 800 nm. The dielectric constant of air is $\varepsilon_{1}=1$. With electromagnetic radiation at the wavelength λ=800 nm, silver has the complex dielectric function $\varepsilon_{Ag}=-30.33+0.81i$, which is calculated using the formula in the ref [11]. Here, we assume that the dielectric medium and Ag are in the region z>0 and z<0, respectively. At boundary 1 (see Figure 1a), x=0. When SPPs propagate along the interface of Ag and a dielectric, the field of SPPs has a component $\propto e^{-i\omega t+i\left(k_{x}x+k_{y}y\right)}$ in the interface plane and exhibits exponential decay away from the interface. The dispersion relations of SPPs can be expressed as [1,11]:

(1) $$k_{spp1}=\frac{\omega }{c}\sqrt{\frac{\varepsilon_{1}\varepsilon_{Ag}}{\varepsilon_{1}+\varepsilon_{Ag}}}$$

(2) $$k_{spp2}=\frac{\omega }{c}\sqrt{\frac{\varepsilon_{2}\varepsilon_{Ag}}{\varepsilon_{2}+\varepsilon_{Ag}}}$$

where $k_{spp1}$ and $k_{spp2}$ represent the wave number of SPPs at the interface of Ag/air and Ag/ma-N 1405, ω indicates the angular laser frequency and *c* is the light velocity in vacuum. The angles of reflection and transmission are more related to the real component of the wave vector rather than its imaginary part, therefore only the real component of $k_{spp}$ is considered for the following calculation and discussion. Combining the above equations with the law of reflection and Snell’s law [70], the angles of reflection ($\theta_{r1}$ and $\theta_{r2}$) and transmission ($\theta_{t1}$ and $\theta_{t2}$) at boundaries 1 and 2 (see Figure 1a) can be written as:

At boundary 1 ($\varepsilon_{1}\to \varepsilon_{2}$),

(3) $$\theta_{r1}=\theta_{i1},$$

(4) $$\theta_{t1}=\arcsin\left(\sqrt{\frac{\varepsilon_{1}\left(\varepsilon_{2}+\varepsilon_{Ag}\right)}{\varepsilon_{2}\left(\varepsilon_{1}+\varepsilon_{Ag}\right)}}\sin\theta_{i1}\right).$$

At boundary 2 ($\varepsilon_{2}\to \varepsilon_{1}$),

(5) $$\theta_{r2}=\arcsin\left(\sqrt{\frac{\varepsilon_{1}\left(\varepsilon_{2}+\varepsilon_{Ag}\right)}{\varepsilon_{2}\left(\varepsilon_{1}+\varepsilon_{Ag}\right)}}\sin\theta_{i1}\right),$$

(6) $$\theta_{t2}=\arcsin\left(\sqrt{\frac{\varepsilon_{2}\left(\varepsilon_{1}+\varepsilon_{Ag}\right)}{\varepsilon_{1}\left(\varepsilon_{2}+\varepsilon_{Ag}\right)}}\sin\theta_{i2}\right)=\theta_{i1}.$$

## 3. Results and Discussion

**Figure 2:**
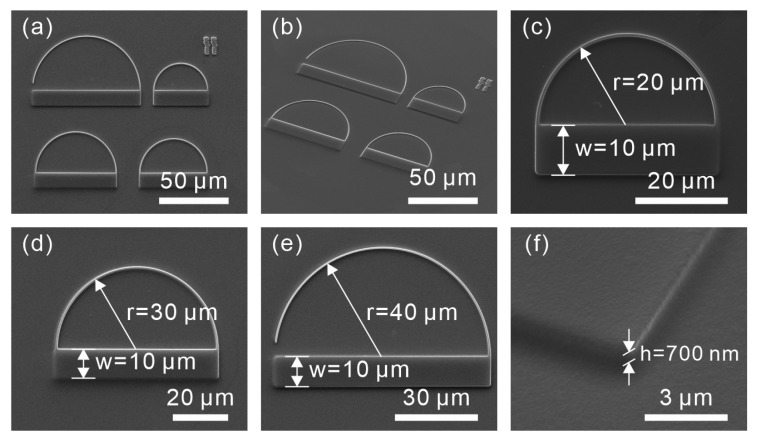
SEM images of the structures fabricated by MPP. (**a**,**b**) SEM images of four structures with different dimensions. (**c**) SEM image of the structure with the radius of curvature r=20 μm. (**d**) SEM image of the structure with r=30 μm. (**e**) SEM image of the structure with r=40 μm. (**f**) The magnified SEM image of the dielectric block.SEM images of the structures fabricated by microscope projection photolithography (MPP)

The proposed structures were fabricated by microscope projection photolithography (MPP). The commercial material ma-N 1405 (microresist technology GmbH) was employed for the fabrication. The refractive index of this material is approximately 1.625 at the wavelength of 800 nm. Figure 2 shows SEM images of fabricated structures obtained with an exposure time of 1000 ms. Structures with radii of r=20, 25, 30 and 40 μm (see Figure 2) were fabricated, respectively. Smooth semicircle ridges and good surface quality dielectric blocks can be observed on each structure. With the exposure duration of 1000 ms, the height of the dielectric block is approximately 700 nm (Figure 2f).

**Figure 3:**
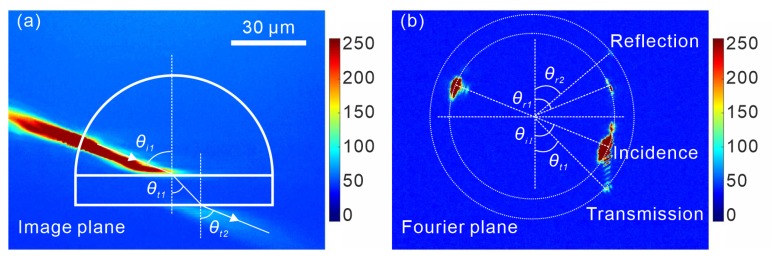
LRM images of the reflection and transmission of SPPs at the boundary of air and the dielectric. The angles of incidence, reflection and transmission, respectively, are indicated by $\theta_{i1}$, $\theta_{r1}$, $\theta_{t1}$ at boundary 1 and $\theta_{i2}$ ($\theta_{i2}=\theta_{t1}$), $\theta_{r2}$, $\theta_{t2}$ at boundary 2. (**a**) Observed image in the image plane. (**b**) Observed image in the Fourier plane.

When excited in the ridge, SPPs propagate along the radial direction at the boundary between Ag and air, with the wave vector $k_{spp1}=\frac{\omega }{c}\sqrt{\frac{\varepsilon_{1}\varepsilon_{Ag}}{\varepsilon_{1}+\varepsilon_{Ag}}}$. When striking onto the dielectric block, the transmitted wave proceeding into the dielectric then propagates at the interface between Ag and the dielectric, with $k_{spp2}\approx \frac{\omega }{c}\sqrt{\frac{\varepsilon_{2}\varepsilon_{Ag}}{\varepsilon_{2}+\varepsilon_{Ag}}}$. The propagation lengths of SPPs along the interfaces of Ag/air and Ag/dielectric were calculated using $L_{spp}=\frac{1}{2k_{spp}^{\prime \prime }}$ [11]. They are approximately 140 μm and 30 μm at the wavelength of 800 nm, respectively. The length of 140 μm is much greater than the obtained maximum radius of the semicircle (40 μm), and 30 μm is larger than the width of the dielectric block (10 μm). Therefore, all the structures could be used for the investigation of the SPP propagation behavior. In this work, the structure with r=40 μm was employed for the experimental investigation for the purpose of ensuring better observation of SPPs in a relatively large space. By installing the sample into the leakage radiation microscopy (LRM) setup and focusing the laser beam onto the semicircle ridge, SPPs can be excited and propagate along the radial direction of the semicircle. Figure 3a is the LRM image of SPPs excited using the proposed structure. Surface plasmon polaritons are split into reflected wave and transmitted wave, when impinging onto the boundary between air and dielectric. The transmitted wave proceeds into the dielectric block and strikes onto boundary 2, where the wave propagates from a dense medium (dielectric) to a thin medium (air). In this case, the wave will be totally reflected when the incident angle $\theta_{i2}⩾\theta_{c}$, with $\theta_{c}=\arcsin\left(\frac{k_{spp1}}{k_{spp2}}\right)=36.73^{\circ }$ indicating the critical angle of total reflection. However, the total reflection does not occur at boundary 2, since the incident wave is exactly the transmitted wave at boundary 1, where SPPs proceed from air into the dielectric block. Based on Snell’s law, the angle of transmission $\theta_{t1}$, which equals the incident angle $\theta_{i2}$, can not exceed $\theta_{c}$. In the image plane (Figure 3a), the transmitted wave out of boundary 2 is visible, while the reflected wave and transmitted wave at boundary 1 are only weakly observable. Consequently, an observation of propagating SPPs in the Fourier plane (Figure 3b) was performed, on which the propagation directions of the waves can be more explicitly visualized.

**Figure 4:**
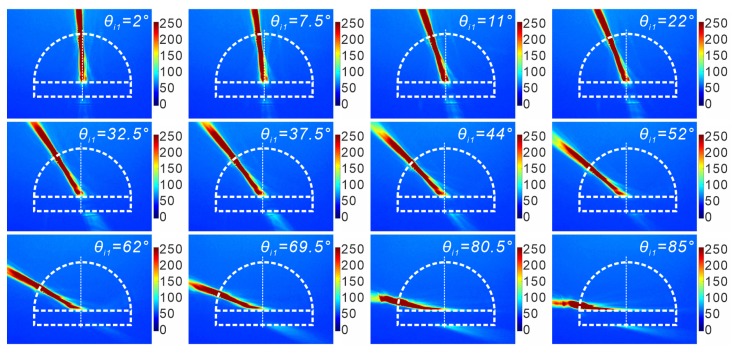
LRM images of the reflection and transmission of SPP waves at the air/dielectric boundary depending on the angle of incidence. The range of the angle of incidence is between $0^{\circ }$ and $90^{\circ }$.

**Figure 5:**
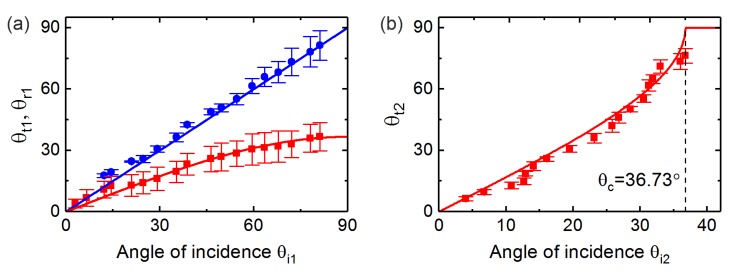
Calculation and experimental measurement of the angles of transmission and reflection with respect to the incident angle. The measurement error of incident angles is within $\pm 2.5^{\circ }$. (**a**) The calculated and measured angles of transmission and reflection at boundary 1. The indications, the blue line (−): calculated $\theta_{r1}$, the blue symbol (•): measured $\theta_{r1}$, the red line (−): calculated $\theta_{t1}$, the red symbol (■): measured $\theta_{t1}$. (**b**) The calculated and measured angle of transmission at boundary 2. The indications, the red line (−): calculated $\theta_{t2}$, the red symbol (■): measured $\theta_{t2}$.

By moving the laser focus onto different positions on the semicircle ridge, the incident angle of SPPs impinging onto boundary 1 can be controlled. Here the reflection and transmission of SPPs with the variation of the incident angle $\theta_{i1}$ (in the range of 0–90${}^{\circ }$) at both boundaries 1 and 2 were observed. Figure 4 presents the images (in the image plane) of SPP propagation with different incident angles. The intensity of SPPs is greatly reduced when the waves hit onto the dielectric block. The reflected waves at those two boundaries and the transmitted wave into the dielectric block are weakly visible. Their existence can only be identified in the Fourier plane. Nevertheless, the transmitted waves out of boundary 2 are always observable in the image plane. Also, an intensity variation of the transmitted wave with respect to $\theta_{i1}$ can be seen. The angles of incidence ($\theta_{i1}$) and transmission ($\theta_{t1}$ and $\theta_{t2}$) can be measured in the figures obtained in the image plane. Additionally, observations of propagating SPPs in the Fourier plane were also performed, from where the angles of reflection ($\theta_{r1}$) for different incident angles are measurable. Here, the angle of reflection at boundary 2 ($\theta_{r2}$) is not taken into consideration due to the invisibility of the corresponding reflected waves within most of the incident angle range. For the purpose of improving the measurement accuracy of those angles, multiple measurements of each single angle ($\theta_{i1}$, $\theta_{r1}$, $\theta_{t1}$ and $\theta_{t2}$) under a fixed wave propagation direction were performed. An average of the multiply measured data for the same angle is taken as the experimental data point (see measured data in Figure 5). The measured data is presented together with an error bar, which is obtained based on the calculated standard deviation. Moreover, the variation of the angles of reflection and transmission with respect to the incident angle was also calculated analytically using Equations (3)–(6). The analytical and measured dependence of the angles of reflection and transmission on the incident angle is presented in Figure 5a indicating the results at boundary 1 and Figure 5b representing the results at boundary 2. Agreement between the analytical results and the experimental results is obtained. It has to be noted that the measurement error mainly results from the irregular form of the propagating SPP waves. Additionally, the weak light intensity for some directions might also affect the measurement results. Furthermore, it will be of significance to simulate and develop an effective approach to quantitatively characterize the intensity variation of propagating SPPs in the proposed structures for better control and optimization of their performance.

## 4. Conclusions

In conclusion, the reflection and transmission effects of SPPs at the boundaries between two different dielectrics are investigated. A special surface metallic structure with micrometer- and partially also nanometer-sized dimensions, which is composed of a dielectric semicircle ridge and a dielectric block, was designed and realized for this investigation. A microscope projection photolithography technique was applied for the fabrication of the proposed structures. The excitation of SPPs and experimental observations of their propagation behavior were realized using leakage radiation microscopy. The variation of the angles of reflection and transmission with respect to the angle of incidence was analytically estimated and experimentally measured. The validity of the law of reflection and Snell’s law in surface plasmon polaritons (SPPs) is confirmed. The integrated structures reported in this work might lay the foundations for novel applications in sensing when functionalized. This will be part of our future work which will aim at measuring the dependence of the SPP behaviors depending on the environmental conditions.

## Abbreviations

The following abbreviations are used in this manuscript:

SPPs	Surface plasmon polaritons
MPP	Microscope projection photolithography
LRM	Leakage radiation microscopy
